# 42.1 BODY AND MIND: CARDIO-METABOLIC AND IMMUNE FUNCTION IN FIRST EPISODE PSYCHOSIS AND COMPARISON WITH CENTRAL NEUROFUNCTIONAL MEASURES

**DOI:** 10.1093/schbul/sby014.174

**Published:** 2018-04-01

**Authors:** Oliver Howes, Toby Pillinger, Katherine Beck, Enrico D’Ambrosio

**Affiliations:** 1 MRC London Institute of Medical Sciences and King’s College London; 2 Institute of Psychiatry, Psychology & Neuroscience, King’s College; 3 King’s College London, MRC Clinical Sciences Centre

## Abstract

**Background:**

People with schizophrenia die on average 15–20 years earlier than the general population, and the mortality gap has grown in recent years. Non-CNS, particularly cardio-metabolic, causes account for the majority of this premature mortality. Abnormalities in the cardio-metabolic and immune system are well established in patients with schizophrenia, but it is unknown if abnormalities are present in people at risk of psychosis and at illness onset prior to antipsychotic treatment, and how they compare to neurofunctional measures. To address this, we conducted a series of meta-analyses and meta-regressions to determine the magnitude and consistency of findings and influence of antipsychotic treatment, exercise and other factors across cardio-metabolic parameters at onset of psychosis.

**Methods:**

We conducted a meta-analysis of peripheral gluco-regulatory, immune and lipid measures and comparison with brain neurofunctional outcomes (including N-acetyl aspartate, gray matter volume and auditory P300 measures) in studies of clinical high risk samples and first episode psychosis, focusing on drug naïve patients. The entire PubMed, EMBase and PsychInfo databases were searched to identify relevant studies. Then we conducted a meta-review statistical comparison of cardio-metabolic and immune function with neurofunctional measures.

**Results:**

35 studies (>1500 patients and controls) were included in the meta-analyses. Interleukin-6 (g=2.2, p=0.013), and TNF-alpha (g=0.94, p<0.01) levels and fasting insulin and post-challenge glucose levels were elevated with moderate-large effect sizes (g=0.4, p=0.01; g=0.6, p=0.007 respectively) and cholesterol and low density lipoprotein levels were reduced (g=-0.2, p=0.005, and g=-0.22, p=0.001 respectively), whilst triglyceride levels were increased (g=0.14, p<0.05). These findings remained significant in drug naïve patients and after adjusting for the influence of a number of potential confounders (including body mass, exercise levels, smoking). N-acetyl aspartate levels in frontal cortex and auditory P300 amplitude (g=0.83, p<0.0001) were significantly altered in first episode patients relative to controls. The median effect sizes for cardiometabolic dysfunction (g=0.41) was comparable to that of the neurofunctional measures (g=0.42, p>0.3). Moreover, the median effect size for immune alterations (g=1.3) was significantly greater than that for neurofunctional measures (p=0.002).

**Discussion:**

We demonstrate that effect sizes for markers of cardio-metabolic and immune disturbance are comparable in magnitude to those for markers of neurofunctional CNS disturbances from the onset of psychosis, suggesting schizophrenia involves multiple organ systems from illness onset. The three main pathoetiological models by which CNS and non-CNS abnormalities may co-occur in schizophrenia will be discussed. The shared genetic and environmental risk architecture between schizophrenia and cardiometabolic disorders suggests common aspects of pathoetiology.

